# Longitudinal effects of rapid maxillary expansion on masticatory muscles activity

**DOI:** 10.4317/jced.53544

**Published:** 2017-05-01

**Authors:** Elena Di Palma, Michele Tepedino, Claudio Chimenti, Gianluca M. Tartaglia, Chiarella Sforza

**Affiliations:** 1 DDS, PhD, Department of Biotechnological and Applied Clinical Sciences, University of L’Aquila, Italy; 2 Professor, Department of Biotechnological and Applied Clinical Sciences, University of L’Aquila, Italy; 3 DDS, PhD, Department of Human Morphology, Functional Anatomy Research Center (FARC), Faculty of Medicine and Surgery, University of Milan, Italy; 4 Professor, Department of Human Morphology, Functional Anatomy Research Center (FARC), Faculty of Medicine and Surgery, University of Milan, Italy

## Abstract

**Background:**

To investigate the modifications induced by rapid maxillary expansion (RME) on the electromyographic (EMG) activities of the anterior temporal and superficial masseter muscles, in patients without pre-treatment EMG alterations.

**Material and Methods:**

Twenty-one patients with unilateral posterior cross-bite selected from the orthodontic department of the University of L’Aquila (Italy), were enrolled. There was no control group in this study since each subject acted as a control of her/himself. Two surface EMG recordings were taken: T0 (before RME) and at T1 (3 month after the end of expansion). To verify the neuromuscular equilibrium, the EMG activities of both right and left masseter and anterior temporal muscles were recorded during a test of maximum clench. EMG indexes were compared by paired Student’s t-test.

**Results:**

In both occasions, all indices showed a good symmetry between the right and left side masticatory muscles. No statistically significant differences were found between the two recordings.

**Conclusions:**

In children without pre-treatment EMG alterations, no variations in standardized muscular activity after RME were found. The treatment did not alter the equilibrium of the masseter and temporal muscles.

** Key words:**Rapid maxillary expansion, electromyography, masticatory muscles.

## Introduction

Rapid maxillary expansion (RME) was proposed for the first time by Angell (1) and later clinically supported by Haas (2). The skeletal modifications induced by this appliance are largely documented in literature (3-5), but no conclusive data about the muscular function were reported.

Muscles represent the functional component of the stomatognathic apparatus, and surface electromyographic (sEMG) examination can be used to investigate their electrical activity (6,7). sEMG is a quick, not invasive and not harmful tool that can provide quantitative data.

The relationship between form and function of the stomatognathic apparatus has been studied in several occasions, and in particular for orthodontic applications (8). Previous investigations also analyzed masticatory muscle activity in patients with maxillary contraction, and in subjects with a normal occlusion, and found several significant differences that supported the priority of treatment of this malocclusion (9,10).

As recently reviewed (10), current reports on the EMG characteristics of the masticatory muscles in patients with crossbite are still controversial. For instance, in some investigations, the activity of masticatory muscles in children with crossbite has been reported to be asymmetrical (i.e., different in the crossed and non crossed sides) both during static (10,11) and dynamic activities (12-14). In contrast, other studies (15,16) found non significant asymmetry indices during rest, maximum voluntary teeth clenching and chewing.

Differences in measurement protocols, as well as in data analysis, may partly explain these contrasting results. Indeed, several authors reported a large variability in sEMG potentials, both in normal controls and in patients with crossbite (9). The great inter individual biological variability can be overcome using standardized EMG potentials, thus removing most of biological and technical noise, and limiting its inherent variability (17). Standardization of sEMG recording, expressing the potentials as a percentage of a maximal voluntary clench, is a practice currently used for the assessment of other body muscles, as repeatedly suggested in literature (18), but seldom used in the assessment of patients with crossbite (19).

Few studies investigated muscular response to expansion therapy, and the results are not always concordant. De Rossi *et al.* (19) found that after RME all the analyzed masticatory muscles increased their activity in comparison with pre-treatment recordings. These authors did not investigate the muscular coordination of their patients, that is the correct relationship between the contractile activities of masticatory muscles, considering both symmetry (paired muscles) and direction of action (for instance, masseter and temporalis). Indeed, considering the altered dental contacts on the crossbite side, it would be interesting to assess if the modification in dental occlusion obtained after a successful RME would result in variations in the neuromuscular coordination.

In contrast, in comparison with pre-treatment recordings, Arat *et al.* (12) found that RME significantly decreased the EMG activities of the anterior temporal and masseter muscles during unilateral chewing. However, 1.5 months after the end of expansion, the EMG activities of both muscles increased and reached pre-treatment values. Throckmorton *et al.* (20) reported similar results: after treatment, maximum mandibular excursions during chewing were unchanged in the crossbite patients, who still maintained a reverse sequence of mastication. A reverse sequence occurs when the mandible first deviates medially and then laterally, thus ensuring overlap of opposing dental occlusal surfaces. In a normal chewing sequence, on the other hand, the mandible deviates laterally, towards the bolus side, and then medially during closure. They concluded that the modifications in the occlusal contacts could not change the central control mechanisms that regulate masticatory activities (20).

Indeed, all orthodontic treatments should be made without altering a normal neuromuscular coordination, and post-treatment EMG activity and symmetry of masseter and temporalis muscles should always remain within normal ranges (8). The aim of the current investigation was to assess the modifications induced by RME on the standardized sEMG activities of the anterior temporal and masseter muscles in a group of children with maxillary contraction who had a good neuromuscular coordination before treatment. In particular, we wanted to investigate if the modifications in occlusal contacts may change the neuromuscular coordination of the masticatory muscles.

## Material and Methods

-Patients

Twenty-one patients (11 girls, 10 boys; mean age 9.8 years, SD 1.6; range 7-12 years), selected from the orthodontic department of the University of L’Aquila (Italy), were studied. The patients were selected from a group of 104 children attending the Clinic according to the following inclusion criteria: transverse maxillary discrepancy, with unilateral posterior cross-bite; Angle Class I occlusion; no articular pain or functional limitations in mandibular movements; no previous or current orthodontic treatment. The project was approved by the Human Research Ethics Committee of the University of L’Aquila (Protocol number 22528) and written informed consent to participate was gathered from parents/legal tutors of all subjects.

Of the 21 subjects, seven were in complete permanent dentition and 14 in mixed dentition; during the study there were no changes in their dental formula. All patients showed a unilateral posterior crossbite with an Angle Class I occlusion, with overjet between 0.5 and 4 mm and overbite between 0 and 4.5 mm.

Before treatment, a sEMG examination verified that all patients had a normal muscular coordination in static conditions, according to Ferrario *et al.* (21): muscular symmetry larger than 80%, potential lateral displacing component smaller than 15%, relative activities of masseter and temporalis muscles within ±15%.

**Figure 1:**
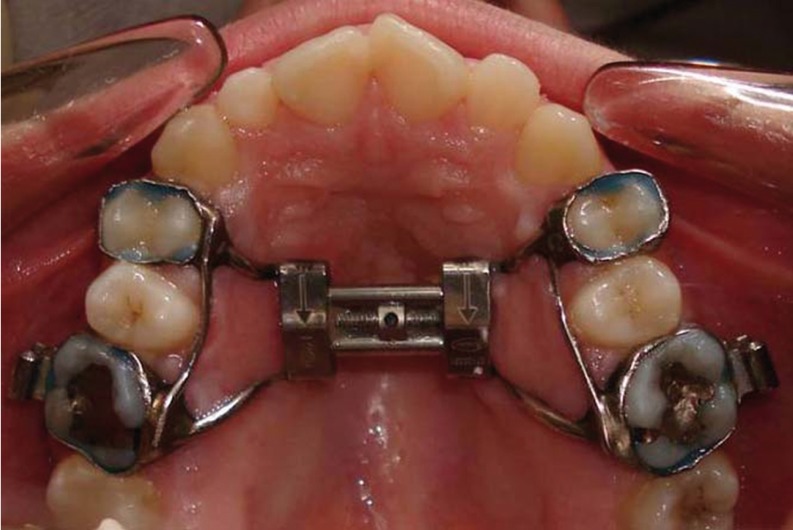
Hyrax appliance bonded to first molars and first premolars.

The subjects were treated with the Hyrax RME appliance (Fig. 1). Hyrax expander is made entirely of stainless steel. Bands are placed on the maxillary first molars and first premolars. Of the 14 patients with mixed dentition, all had first premolars erupted, allowing to use the same appliance design in all subjects. The expansion screw is localized in the palate in close proximity to the palatal contour. The rapid expander is the most used therapeutic appliance to resolve a transversal deficit of the maxilla. It is an orthopaedic appliance that acts on the skeletal bases and applies heavy forces. These forces do not act only on the midpalatal suture, but also on the circummaxillary ones. Therefore, modifications can be obtained in all spatial planes. The appliance was activated twice a day (0.25 mm per activation) until overcorrection of the posterior crossbite was achieved. The average treatment period was 3 weeks.

There was no control group in this study since each subject acted as a control of her/himself.

-sEMG analysis

To evaluate the muscular equilibrium in static conditions, sEMG analysis of right and left masseter and anterior temporalis muscles was performed in all patients (17,21) in two occasions: before rapid palatal expansion (T0) and 3 months after the end of expansion (T1). All measurements were performed without the palatal expansor (at T0, before cementing it; at T1, the day after its removal).

All patients were submitted to the EMG exam only after obtaining written informed consensus from parents/ legal tutors. Verbal consensus was also obtained by all children.

**Figure 2:**
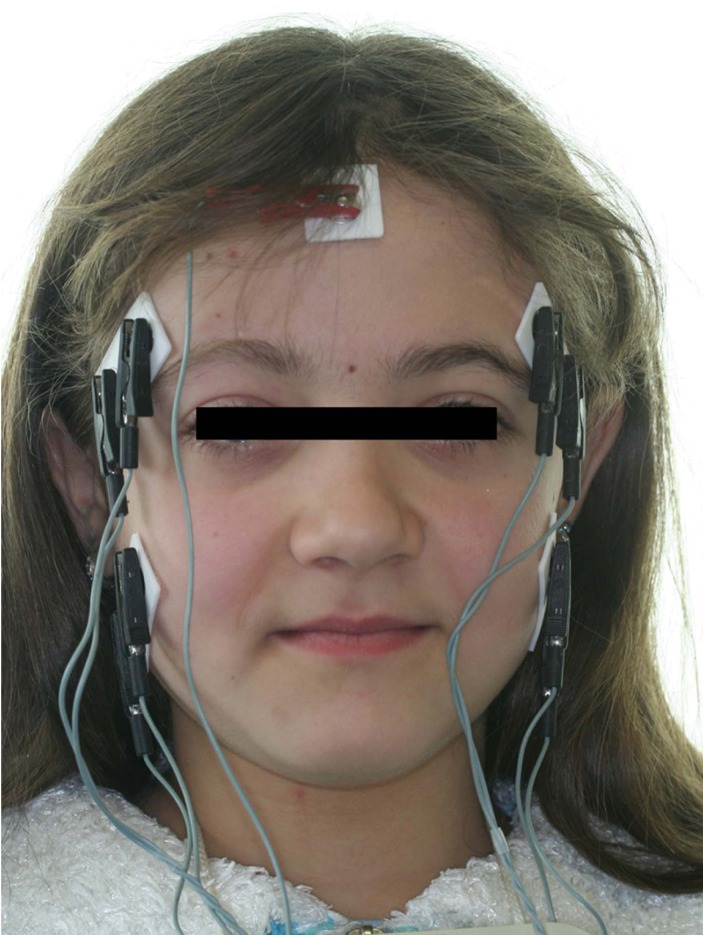
Positioning of the electrodes according to the SENIAM recommendation.

To reduce impedance, the skin was carefully cleaned before the electrode placement, and recordings were performed 5-6 minutes later, allowing the conductive paste to adequately moisten the skin surface. During testing disposable silver/silver chloride bipolar electrodes with a diameter of 10 mm and an interelectrode distance of 21±1 mm (Duo-Trode; Myo-Tronics Inc., Seattle, WA, USA) were used. The reference electrode was positioned on the forehead. The electrodes were located according to the recommendations of SENIAM (Surface EMG for Non-Invasive Assessment of Muscles (22) (Fig. 2).

The EMG activity was recorded using an instrument that recorded, amplified, digitized and filtered the analogical EMG signal (21) (De Gotzen srl, Legnano, Milano, Italy). The analog EMG signal was amplified and digitized (gain 150, peak-to-peak input range 28 mV, that is ±14 mV, 12 b resolution, 2230 Hz A/D sampling frequency, theoretical resolution 16 lV) using a differential amplifier with a high common mode rejection ratio (CMRR = 105 dB in the range 0–60 Hz, input impedance 10 GX), and filtered (analogue filtering: lowpass filter with a bandwidth in the frequency range 0–580 Hz; digital filtering: range 30–400 Hz; band-stop for common 50 Hz interference with a notch filter, approximate range 47– 53 Hz). Very low frequency (<10 Hz) artefacts were limited by the use of the reference electrode (forehead). Previous bench assessments found that the peak-to-peak input range measured by the instrument was larger than the offset values obtained by the surface electrodes positioned according to the current protocol. The signals were averaged over 25 ms, with muscle activity assessed as the root mean square (RMS) of the amplitude (unit: l V). EMG signals were recorded for further analysis.

Two EMG recordings were made in each session (21):

1) standardization recording: 5 seconds maximum voluntary clench (MVC) performed on two 10-mm thick cotton rolls positioned between the mandibular posterior teeth;

2) experimental recording: 5 seconds MVC performed in maximum occlusion without cotton rolls.

The 3 seconds with the most stable EMG signal were automatically selected by the software in both cases.

For each patient, the EMG potentials of the analyzed muscles recorded during the MVC tests were expressed as percent of the mean potential recorded during the standardization test (MVC on the cotton rolls), unit: μV/μV x 100. All subsequent calculations were made with the standardized potentials. Relative percentage EMG values should be affected only by the occlusal surfaces, because this kind of standardization should annul the variability caused by skin and electrode impedance, electrode positioning (17,23).

-sEMG indices of neuromuscular coordination

For each patient, several EMG indexes (symmetry, torque, relative activity) were computed. Besides, the total standardised muscle electric activity developed by the four investigated muscles during the MVC was obtained

To assess muscle symmetry, within each subject the EMG waves of paired muscles were compared by computing a percentage overlapping coefficient (POC, unit: %).

The total standardised electric activity developed by the four investigated muscles during the MVC was computed as the average integrated areas of the masseter and temporalis EMG potentials over time (unit: µV/µV s %). The value developed during the MVC on occlusal surface was obtained as a percentage of the muscular activity developed during the cotton roll clenching (21).

To test the error of the method, the measurements were repeated in 10 randomly selected patients. For all EMG variables, the intraclass correlation coefficients were larger than 0.62, showing a good accuracy of the measurements, without random errors (paired Student’s t test, *p* > 0.05).

Pre- (T0) and post- (T1) expansion data were compared with Student’s t test for paired samples with a predefined significance level of 5% (*p*<0.05).

## Results

The repeatability of sEMG recordings of masseter and anterior temporalis muscles was tested in FARC laboratory and in Orthodontic Department of University of L’Aquila (21).

As reported in  Table 1, the statistical analysis did not show statistically significant differences in the electric standardized activity of the masticatory muscles (masseter and anterior temporal) between the EMG recordings carried out before and after RME (Student’s t test, *p*>0.05). In all patients, the standardized distribution of the electric activity of the anterior temporal and masseter muscles did not vary after RME.

**Table 1:**
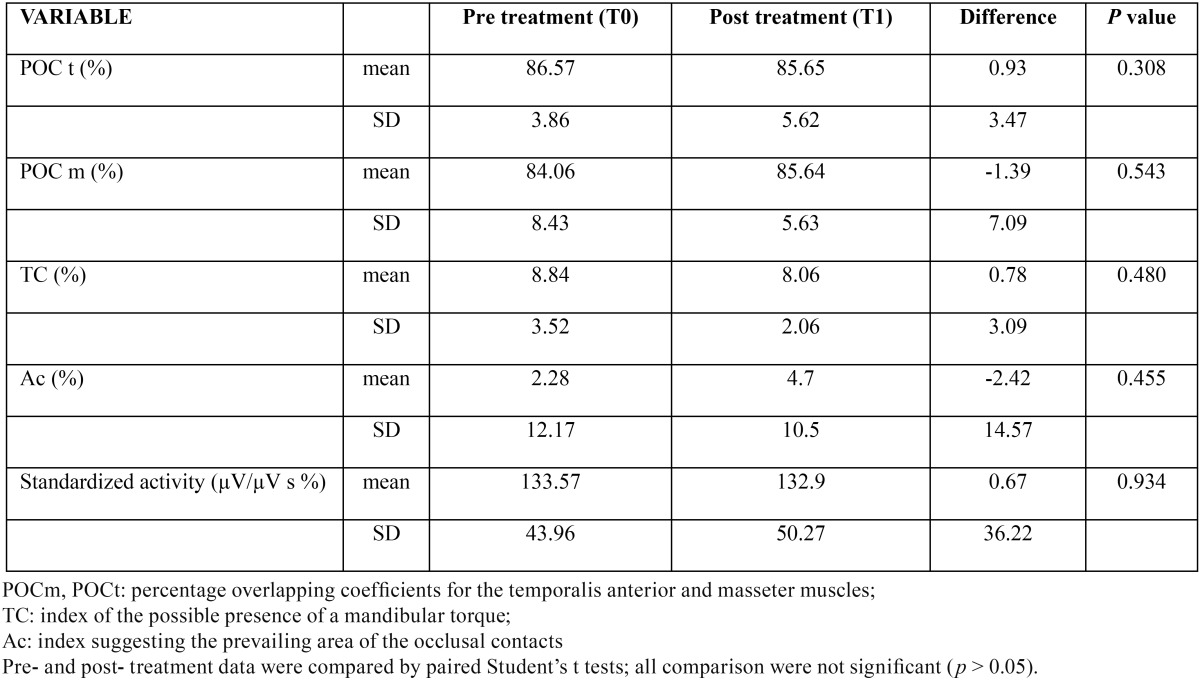
EMG indices in 21 patients with maxillary contraction: differences between before and after a rapid maxillary expansion.

Before treatment, all children had a good muscular equilibrium, with EMG indices within reference values (21). After treatment, all EMG indices remained within reference values, showing a good symmetry between the right and left side masticatory muscles, without any mandibular torque, and with a correct distribution of the occlusal centre of pressure.

## Discussion

The electric activity of the masticatory muscles in subjects with normal occlusion and in patient with crossbite has been investigated in several occasions, but contradicting results had been reported so far (11,24).

Andrade *et al.* (11) found that during teeth clenching the masseter muscle was significantly more active on the crossbite side than on the normal side, while no differences were observed at rest and in the temporalis anterior muscle. In contrast, other authors found that the posterior temporalis of the crossbite side showed higher EMG activity than the contralateral muscle at rest and in clenching, whereas the ipsilateral anterior temporalis was less active than the contralateral muscle at rest (24).

Other studies (25), on the other hand, showed that the posterior temporal of the crossed side was less active than the contralateral muscle. The authors hypothesized that this asymmetry in the posterior temporal was probably a result of the functional mandibular shift that exists in patients with unilateral crossbite; therefore, they considered that the posterior temporal has a positioning and stabilising function on the mandible. In the same study, the authors showed that the anterior temporal of the contralateral side was more active than that of the crossed side in subjects with crossbite than in subjects with a normal occlusion.

According to the current findings, children with crossbite but with a good neuromuscular coordination before treatment can have a fair neuromuscular adaptability after RME. Indeed, the neuromuscular system can be stable even with a crossed occlusion: quality of contacts is more important than quantity (6).

Arat *et al.* (12) showed that 1.5 months after maxillary expansion, the EMG potentials returned similar to the pre-treatment values. The current findings showed that the muscular coordination was within normal values 3 months after RME, with no statistically significant differences in the symmetric distribution of the electric activity of right and left masseter and temporal muscles.

The POC, TC and Ac indexes of the current EMG study emphasize the role occlusion has on the neuromuscular equilibrium, a correlation widely proven by numerous studies (26).

POC is an index of the symmetric distribution of muscular activity as determined by occlusion. It ranges between 0 (no symmetry) and 100% (perfect symmetry); values higher than 85% are considered normal (17). POC index was calculated for each couple of homologous muscles (masseter POC, temporalis POC).

The activity index (Ac) was calculated to compare the muscular activities of masseter and temporalis muscles; the index informs about the prevailing area of the contacts, and thus about the principal occlusal centre of pressure (27). When the standardized EMG potentials are not balanced between the two analyzed masticatory muscles, the occlusal centre of pressure (clench on the occlusal surfaces as compared to clench on the cotton rolls) might be displaced onwards (temporalis prevalent) or backwards (masseter prevalent). Ac ranges from -100% (temporalis muscle prevalence) to +100 (masseter muscle prevalence) (28). A negative activity coefficient has already been reported to be determined from dental contacts in the anterior arch, with a larger load on the temporomandibular joint (28).

Because an unbalanced contractile activity of contralateral masseter and temporalis muscles, i.e. right temporalis and left masseter, might cause a potential lateral displacing component, the Torque Coefficient (TC) was computed (17). TC ranges between 0% (well comparable standardized masseter and temporalis potentials) and 100% (unbalanced standardized masseter and temporalis potentials). When the index is higher than 10%, there is a muscular latero-deviant couple that may push the mandible towards left or right.

The limited number of analyzed patients does not allow making conclusive statements on this topic, even if the longitudinal assessments and the use of standardized EMG potentials enhance the significance of the current findings. As in previous reports on the same topic and with a short observation period (12), we did not include a control group because each subject acted as a control of her/ himself.

The same Hyrax expander with four bands was used for all patients, because even the 14 patients with mixed dentition of our sample had upper first premolars erupted. This means that some of these patients had premature exfoliation of upper first deciduous molars, which usually occurs around 9-10 years of age; all these subjects were girls, which are known to have permanent teeth erupting earlier than boys (29). However, since no changes in the dental formula of the subjects were observed during the observation period, no effect on the results of the present study can be presumed.

One of the limitation of the current study, as of other studies (12,19,20), is the lack of a medium- and long-term follow up: patients were analyzed only up to 3 months after the end of RME. In contrast, Ferrario *et al.* (8) followed up their patients for 12 months, with monthly sEMG examinations made during periodic dental controls.

Long-term studies with larger samples are required to determine if alterations of the sEMG activity occur when the malocclusion remains untreated, to elucidate if early treatment is useful or not, and to evaluate the cost-benefit rate of an early intervention. Furthermore, the current study investigated only the static neuromuscular coordination of crossbite patients, and further investigations should include also dynamic assessments (for instance, chewing) (12,13).

Additional information may be obtained assessing the jaw movements by motion analysis instruments eventually coupled with surface EMG recordings (14,20,30).

## Conclusions

Within the limitations of the current study, RME represents, therefore, an efficient therapeutic method not only from the dental and skeletal points of view, but also from the functional side, because the treatment did not alter the equilibrium of the masseter and temporal muscles. In the analysed children, the new occlusal condition was well compatible with symmetric muscular activities during clenching.

The preliminary results of this study indicate that the masticatory musculature can well adapt to RME, at least in children with a good muscular coordination before treatment.

## References

[1] Angell EH (1860). Treatment of irregularity of the permanent or adult teeth. Dent Cosmos.

[2] Haas AJ (1970). Palatal expansion: just the beginning of dentofacial orthopedics. Am J Orthod.

[3] McNamara JA, Baccetti T, Franchi L, Herberger TA (2003). Rapid maxillary expansion followed by fixed appliances: a long-term evaluation of changes in arch dimensions. Angle Orthod.

[4] Sandikçioğlu M, Hazar S (1997). Skeletal and dental changes after maxillary expansion in the mixed dentition. Am J Orthod Dentofac Orthop.

[5] Chung CH, Font B (2004). Skeletal and dental changes in the sagittal, vertical and transverse dimensions after rapid palatal expansion. Am J Orthod Dentofac Orthop.

[6] Di Palma E, Gasparini G, Pelo S, Tartaglia GM, Chimenti C (2009). Activities of masticatory muscles in patients after orthognathic surgery. J Craniomaxillofac Surg.

[7] De Felício CM, Sidequersky FV, Tartaglia GM, Sforza C (2009). Electromyographic standardized indices in healthy Brazilian young adults and data reproducibility. J Oral Rehabil.

[8] Ferrario VF, Marciandi PV, Tartaglia GM, Dellavia C, Sforza C (2002). Neuromuscular evaluation of post-orthodontic stability: an experimental protocol. Int J Adult Orthodon Orthognath Surg.

[9] Alarcón JA, Martín C, Palma JC (2000). Effect of unilateral posterior crossbite on the electromyographic activity of human masticatory muscles. Am J Orthod Dentofacial Orthop.

[10] Andrade AS, Gameiro GH, Derossi M, Gavião MB (2009). Posterior crossbite and functional changes. A systematic review. Angle Orthod.

[11] Andrade AS, Gavião MB, Derossi M, Gameiro GH (2009). Electromyographic activity and thickness of masticatory muscles in children with unilateral posterior crossbite. Clin Anat.

[12] Arat FE, Arat ZM, Acar M, Beyazova M, Tompson B (2008). Muscular and condylar response to rapid maxillary expansion. Part 1: electromyographic study of anterior temporal and superficial masseter muscles. Am J Orthod Dentofacial Orthop.

[13] Ferrario VF, Sforza C, Serrao G (1999). The influence of crossbite on the coordinated electromyographic activity of human masticatory muscles during mastication. J Oral Rehabil.

[14] Piancino MG, Farina D, Talpone F, Merlo A, Bracco P (2009). Muscular activation during reverse and non-reverse chewing cycles in unilateral posterior crossbite. Eur J Oral Sci.

[15] Alarcón JA, Martín C, Palma JC, Menéndez-Núñez M (2009). Activity of jaw muscles in unilateral cross-bite without mandibular shift. Arch Oral Biol.

[16] Lenguas L, Alarcòn JA, Venancio F, Kassem M, Martin C (2012). Surface electromyographic evaluation of jaw muscles in children with unilateral crossbite and lateral shift in the early mixed dentition. Sexual dimorphism. Med Oral Patol Oral Cir Bucal.

[17] Ferrario VF, Sforza C, Colombo A, Ciusa V (2000). An electromyographic investigation of masticatory muscles symmetry in normo-occlusion subjects. J Oral Rehabil.

[18] Armijo-Olivo S, Gadotti I, Kornerup M, Lagravère MO, Flores-Mir C (2007). Quality of reporting masticatory muscle electromyography in 2004: a systematic review. J Oral Rehabil.

[19] De Rossi M, De Rossi A, Hallak JE, Vitti M, Regalo SC (2009). Electromyographic evaluation in children having rapid maxillary expansion. Am J Orthod Dentofacial Orthop.

[20] Throckmorton GS, Buschang PH, Hayasaki H, Pinto AS (2001). Changes in the masticatory cycle following treatment of posterior unilateral crossbite in children. Am J Orthod Dentofacial Orthop.

[21] Ferrario VF, Tartaglia GM, Galletta A, Grassi GP, Sforza C (2006). The influence of occlusion on jaw and neck muscle activity: a surface EMG study in healthy young adults. J Oral Rehabil.

[22] Hermens HJ, Freriks B, Disselhorst-Klug C, Rau G (2000). Development of recommendations for SEMG sensors and sensor placement procedures. J Electromyogr Kinesiol.

[23] Castroflorio T, Farina D, Bottin A, Piancino MG, Bracco P (2005). Surface EMG of jaw elevator muscles: effect of electrode location and inter-electrode distance. J Oral Rehabil.

[24] Troelstrup B, Möller E (1970). Electromyography of the temporalis and masseter muscles in children with unilateral crossbite. Scand J Dent Res.

[25] Ingervall B, Thilander B (1975). Activity of temporal and masseter muscles in children with a lateral forced bite. Angle Orthod.

[26] Di Palma E, Gasparini G, Pelo S, Tartaglia GM, Sforza C (2010). Activities of masticatory muscles in patients before orthognathic surgery. J Craniofac Surg.

[27] Naeije M, McCarroll RS, Weijs WA (1989). Electromyographic activity of the human masticatory muscles during submaximal clenching in the inter-cuspal position. J Oral Rehabil.

[28] Ferrario VF, Sforza C (1994). Biomechanical model of the human mandible in unilateral clench: distribution of temporomandibular joint reaction forces between working and balancing sides. J Prosthet Dent.

[29] Hernández M, Espasa E, Boj JR (2008). Eruption chronology of the permanent dentition in Spanish children. J Clin Pediatr Dent.

[30] De Felício CM, Mapelli A, Sidequersky FV, Tartaglia GM, Sforza C (2013). Mandibular kinematics and masticatory muscles EMG in patients with short lasting TMD of mild-moderate severity. J Electromyogr Kinesiol.

